# AQP4‐IgG positive paraneoplastic NMOSD: A case report and review

**DOI:** 10.1002/brb3.2282

**Published:** 2021-09-14

**Authors:** Manqiu Ding, Yue Lang, Li Cui

**Affiliations:** ^1^ Department of Neurology The First Hospital of Jilin University Changchun Jilin Province P. R. China

**Keywords:** AQP4, NMOSD, paraneoplastic

## Abstract

**Introduction:**

Neuromyelitis optica spectrum disorder (NMOSD; also known as Devic syndrome) is a clinical syndrome of central nervous system characterized by immune mediated attacks of acute optic neuritis and myelitis. Paraneoplastic neurological syndrome is a group of nervous system disorders resulting from the remote immune effects of malignant neoplasm. NMOSD occurs mostly in young people, and tumor is not a common cause, especially recurrent tumor.

**Methods:**

We reported a case of a 59‐year‐old man who developed anti‐aquaporin‐4 IgG positive longitudinally extensive myelitis. We also summarized and analyzed previously reported cases of paraneoplastic NMOSD.

**Results:**

Among these 43 patients, 88.4% patients are female. The largest number of patients is between 60 and 69 years old. Breast cancer and lung cancer are the most common types. The most common lesions were located in the cervicothoracic region with patchy gadolinium enhancement. The existing treatment can only delay rather than stop the progress of the disease.

**Conclusion:**

It is necessary to perform tumor screening in patients with NMOSD, especially patients over 50 years.

## Abstract 2

Wereported a rare case of AQP4‐IgG positive paraneoplastic NMOSD with recurrent rectal cancer. We summarized the previously reported cases of paraneoplastic NMOSD from demographics, clinical features, neuroimaging findings, laboratory examination, therapy, outcome, and other aspects and highlighted the necessity to perform tumor screening in patients with NMOSD, especially patients over 50 years. We analyzed the pathogenesis of AQP4‐IgG positive paraneoplastic NMOSD and discussed the phenomenon of paraneoplastic NMOSD with intractable hyponatremia.

## 1 INTRODUCTION

In 1894, Devic and Gault pioneeringly described the clinical characteristics of neuromyelitis optica (derived from neuro‐myélite optique aiguë)—optic neuritis (ON) and acute transverse myelitis, which makes it also known as Devic syndrome (Devic, 1894). With further understanding of clinical, radiological, and immunological characteristics of neuromyelitis optica spectrum disorder (NMOSD), especially aquaporin‐4 (AQP4) antibody, NMOSD as an independent central nervous system disease has attracted more and more attention (Lennon et al., 2004). Longitudinally extensive myelitis (LETM), ON, and/or bouts of intractable vomiting and hiccoughs (area postrema syndrome) are classic presentations of paraneoplastic NMOSD (Grativvol et al., 2018). NMOSD could be divided into AQP4‐IgG positive NMOSDs (more than 80%), MOG‐IgG positive NMOSD (10%–40% of AQP4‐IgG negative NMOSD), and seronegative NMOSD (the other AQP4‐IgG negative NMOSD) according to the types of antibody (Jarius et al., 2020). Many factors, including vaccinations, infection, systemic autoimmune diseases, cancer, and so on, have been considered to be related to the morbidity of NMOSD but the specific mechanism is not yet fully understood (Wingerchuk et al., 2007). The paraneoplastic neurological syndrome is complex and may affect many parts of peripheral or central nervous system and in different forms (Yi & Park, 2020). With the increase of AQP4‐positive paraneoplastic NMOSD cases being reported, whether the occurrence of NMOSD is related to the origin of tumor, and whether cancer screening is needed when NMOSD is suspected have become the concern of clinicians. The central theory of autoimmunity postulates that “onconeural antigen(s)” expressed by tumor have the same characteristics as the antigens expressed by neurons. This allows antibodies produced against tumor surface antigens to attack neurons, leading to paraneoplastic neurological symptoms. However, some studies have shown that these “onconeural antigen(s)” only have a weak immune effect on the central nervous system, which is not enough to cause serious damage, so the underlying mechanism of paraneoplastic neurological diseases still remain poorly understood (Dropcho, 2005). However, the early diagnosis of paraneoplastic NMOSD is of great significance for the prognosis of the disease. Herein, we reported a case of AQP4‐positive paraneoplastic NMOSD with recurrent rectal cancer and performed a retrospective analysis of previously reported cases and review articles.

## 2 CASE PRESENTATION

A 59‐year‐old male patient presented to hospital with recurrent paroxysmal pain in bilateral upper arms for 4 months and aggravated with right leg weakness for 4 days. He has a history of rectal cancer (moderately differentiated adenocarcinoma) for 2 years and underwent surgery and six cycles of systemic chemotherapy. On the admission neurological examination, the patient was conscious and oriented, with fluent speech. Except for the failure of bilateral eyelid to be closed completely, results of the other cranial nerve examination were normal. Motor examination revealed severe weakness in right legs with grade 5 in the upper extremities and left lower extremity and grade 1 in the right lower extremity (Medical Research Council). Muscle tone was normal and there was a slight hyporeflexia in his right lower extremity. The sensory examination showed hypoalgesia at the left side of body below T1 level and hypoesthesia at left toe. Babinki and Chaddock's reflex was positive bilateral. There were no signs of meningeal irritation.

**FIGURE 1:**
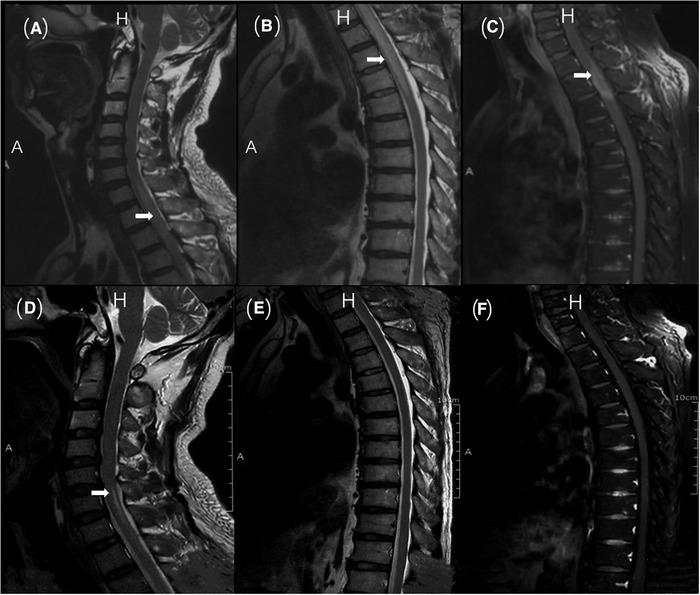
**Spine cord MRI of the patient**. The spine cord MRI (A–C) shows continuous T2 hyperintensity lesion extending from C6 to T5, with focal patchy gadolinium enhancement at T1–T3. Sagittal T2 image of the cervical spine (A). Sagittal T2 image of the thoracic spine (B). Sagittal T1 image of the thoracic spine postinfusion of gadolinium, the arrowhead indicates the area of gadolinium enhancement (C). A total recovery of the spinal cord swelling and a few T2 hyperintensity with no enhancement 1 month later (D–F). Sagittal T2 image of the cervical spine (D). Sagittal T2 image of the thoracic spine (E). Sagittal T1 image of the thoracic spine postinfusion of gadolinium (F). A indicates anterior and H indicates head

**FIGURE 2:**
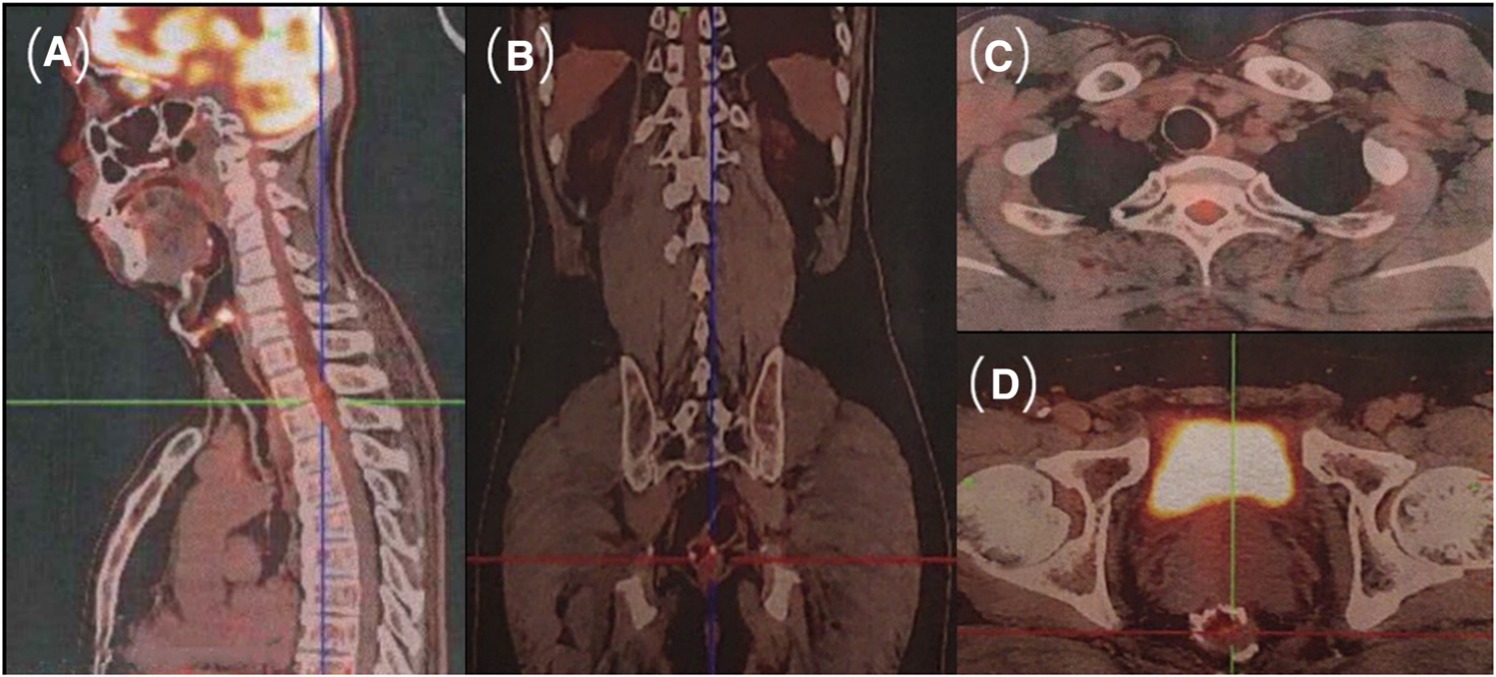
**Spinal cord and rectal PET‐CT of the patient**. PET‐CT reveals a stripe fluorine‐18‐fluorodeoxyglucose (FDG)‐avid region at the T1‐T3 level (A/C). Sagittal PET/CT image of cervicothoracic region (A). Axial PET/CT image of thoracic region (C). PET‐CT shows rectal wall thickening with hypermetabolism in rectal anastomotic area (B/D)

Brain magnetic resonance imaging (MRI) did not show any new lesions. Spine MRIs showed continuous T2 hyperintensity extending from C6 to T5, with focal patchy gadolinium enhancement at T1–T3 (Figure 1). A whole‐body PET‐CT (positron emission tomography) showed a stripe fluorine‐18‐fluorodeoxyglucose (FDG)‐avid region in the spinal canal at the T1–T3 level with the maximum standardized uptake value (SUVmax) of 5.8 (Figure 2). PET‐CT also showed the rectal wall thickening and hypermetabolism beside the anastomotic line, which was suspected to be tumor recurrence (Figure 2). The lumbar puncture was performed in the patient for cerebrospinal fluid (CSF) analysis and the results were given in Table 1. Protein, IgG, white blood cells in CSF were slightly higher than the normal range that did not have specific diagnostic value. We further tested AQP4 antibodies in CSF and serum by live CBA assay, the AQP4‐IgG was 1:100+ positive in CSF and 1:10+ positive in serum. In addition, he has a refractory hyponatremia with serum sodium concentration ranging from 129.3 to 135.8 mmol/L.

**TABLE 1:** The results of the CSF analyses of our case

	Case	Reference value
CSF pressure	80 mmHg	80–170 mmH_2_O
AQP4 IgG (CBA)	1:100+	
WBC	23 × 10^6^	0–8 × 10^6^
Lymphocyte	91%	–
Neutrophil	5%	–
Monocyte	4%	–
RBC	0	
Protein	0.76 g/L	0.15–0.45 g/L
Pandy test	Positive	Negative
Chloride	122.4 mmol/L	119–129 mmol/L
Glucose	4.38 mmol/L	2.3–4.1 mmol/L
IgG	76.4 mg/L	0–34 mg/L
IgG Index	0.46	–
OB	Negative	Negative
*Q*_A1b_	12.12 × 10^−3^	(*Q*_Alb_ < 7 × 10^−3^, 45 years old, *Q*_Alb_ < 8 × 10^−3^, 60 years old, *Q*_Alb_ < 9 × 10^−3^, 75 years old)
Reibergram histogram (IgG, IgA, IgM)	Simple blood–brain barrier dysfunction	–
Paraneoplastic antibody panel (Yo, Hu, Ri, CV2, Amphiphysin, Ma1, Ma2, SOX1, Tr, Zic4, GAD65, PKCγ, Recoverin, Titin)	Negative	Negative
TB‐Ab	Negative	Negative

Abbreviations: CSF, cerebrospinal fluid; *Q*_A1b_, ratio of Alb concentration in CSF and serum; RBC, red blood cell; TB‐Ab, tubercle bacillus antibody; WBC, white blood cell

Then a 5‐day immunomodulatory therapy with gamma globulin (intravenous immunoglobulin [IVIG]) was started, however, the patient continued to deteriorate and reached the most serious condition on the 10th day of admission. Motor examination showed that the strength of both upper extremities was grade 5, the strength of left lower extremity was grade 2, and the strength of right lower extremity was grade 0 (Medical Research Council). After that he received therapy with high‐dose glucocorticoid for 12 days (methylprednisolone, 1000 mg for 3 days, 500 mg for 3 days, 240 mg for 3 days, and 120 mg for 3 days). After these series of treatment, the serum AQP4 antibody was still positive, but the titer was significantly lower than before. When the patient was discharged, the muscle strength of both upper extremities was grade 5, the left lower extremity was grade 3, and the right lower extremity was grade 0, which was better than the most serious condition but the patient was still unable to live without the wheelchair. The immunotherapy of this patient was maintained with oral steroid after discharge. After 1 month of follow‐up, this patient made a relatively good recovery with both clinical and radiological improvement. Motor examination showed that the strength of left lower extremity gradually recovered to grade 5 and grade 1 in the right lower extremity. Cervical and thoracic MRI revealed a total recovery of the spinal cord swelling and a few T2 hyperintensity with no enhancement (C6–C7 and T2–T3; Figure 1). Now the immunotherapy was continued with 40 mg oral steroid per day. Then, a 4‐month immunosuppressive therapy was performed with i.v. 1000 mg cyclophosphamide once per month.

Our study was approved by the Ethics Committee of The First Hospital of Jilin University.

## 3 METHODS

Two independent reviewers conducted an independent search about the paraneoplastic NMOSDs up to November 2020 on PubMed databases using predefined medical subject headings (MeSH), such as paraneoplastic, cancer, and NMOSD. All studies reporting cases of AQP4 positive paraneoplastic NMOSD were included in Table S1 and used for the quantitative analysis (Table 2; Al‐Harbi et al., 2014; Annus et al., 2018; Armağan et al., 2012; Baik et al., 2018; Bernard‐Valnet et al., 2019; Cai et al., 2016; De Santis et al., 2009; Etemadifar et al., 2019; Fang et al., 2019; Figueroa et al., 2014; Frasquet et al., 2013; Jin et al., 2019; Kitazawa et al., 2012; Kon et al., 2017; Liao et al., 2019; Mahale et al., 2019; Mueller et al., 2008; Nakayama‐Ichiyama et al., 2011; Pittock & Lennon, 2008; Verschuur et al., 2015; Wang et al., 2015; Wiener et al., 2018; Yang et al., 2014; Yi & Park, 2020; Yuan et al., 2019). Finally, 43 cases from 26 articles were included (Table S1; Al‐Harbi et al., 2014; Annus et al., 2018; Armağan et al., 2012; Baik et al., 2018; Bernard‐Valnet et al., 2019; Cai et al., 2016; De Santis et al., 2009; Etemadifar et al., 2019; Fang et al., 2019; Figueroa et al., 2014; Frasquet et al., 2013; Jin et al., 2019; Kitazawa et al., 2012; Kon et al., 2017; Liao et al., 2019; Mahale et al., 2019; Mueller et al., 2008; Nakayama‐Ichiyama et al., 2011; Pittock & Lennon, 2008; Verschuur et al., 2015; Wang et al., 2015; Wiener et al., 2018; Yang et al., 2014; Yi & Park, 2020; Yuan et al., 2019).

**TABLE 2:** Main demographic data, clinical characteristics, laboratory, and imaging results in described AQP4‐positive paraneoplastic NMOSD

Demographic data	
Mean age, years	50
Female, n (%)	38 (88.4%)
Clinical characteristics	
LETM, n (%)	35 (81.4%)
ON, n (%)	20 (46.5%)
Area postrema symptom, n (%)	10 (23.3%)
Location of neuroimaging	
Brain, n (%)	8 (27.6%)
Cervical, n (%)	18 (62.1%)
Thorax, n (%)	17 (58.6%)
Lumbar, n (%)	1 (3.4%)
Conus, n (%)	2 (6.9%)
CSF investigations	
Increased CSF protein, n (%)	14 (58.3%)
Increased white cells, n (%)	10 (40%)
Oligoclonal bands, n (%)	6 (33.3%)
Onset CSF AQP4 positive, n (%)	5 (11.6%)
Serological status	
Onset serum AQP4 positive, n (%)	100%
AQP4 positive in tumor tissue	7 (63.3%)
Accomplished cancer	
Breast cancer, n (%)	10 (22.2%)
Lung cancer, n (%)	6 (13.3%)
Time from NMOSD to cancer diagnosis	
Before cancer	26 (60.5%)
After cancer	17 (39.5%)
Treatment	
IVMP, n (%)	28 (100%)
IVIGA, n (%)	6 (21.4%)
PE, n (%)	7 (25%)
Surgery, n (%)	8 (28.6%)
Azathioprine, n (%)	7 (25%)
Tacrolimus, n (%)	1 (3.6%)
Rituximab, n (%)	3 (10.7%)
Oral steroid, n (%)	10 (35.7%)
Chemotherapy, n (%)	11 (39.3%)
Response to treatment	
CR, n (%)	5 (17.2%)
PR, n (%)	20 (69%)
Die, n (%)	4 (13.8%)

Abbreviations: AQP4, aquaporin‐4; CR, complete recovery; CSF, cerebrospinal fluid; IVIGA, intravenous immunoglobulin; IVMP, intravenous methylprednisolone; LETM, longitudinally extensive myelitis; NMOSD, neuromyelitis optica spectrum disorders; ON, optic neuritis; PE, plasma exchange; PR, partial recovery.

## 4 RESULTS

**FIGURE 3:**
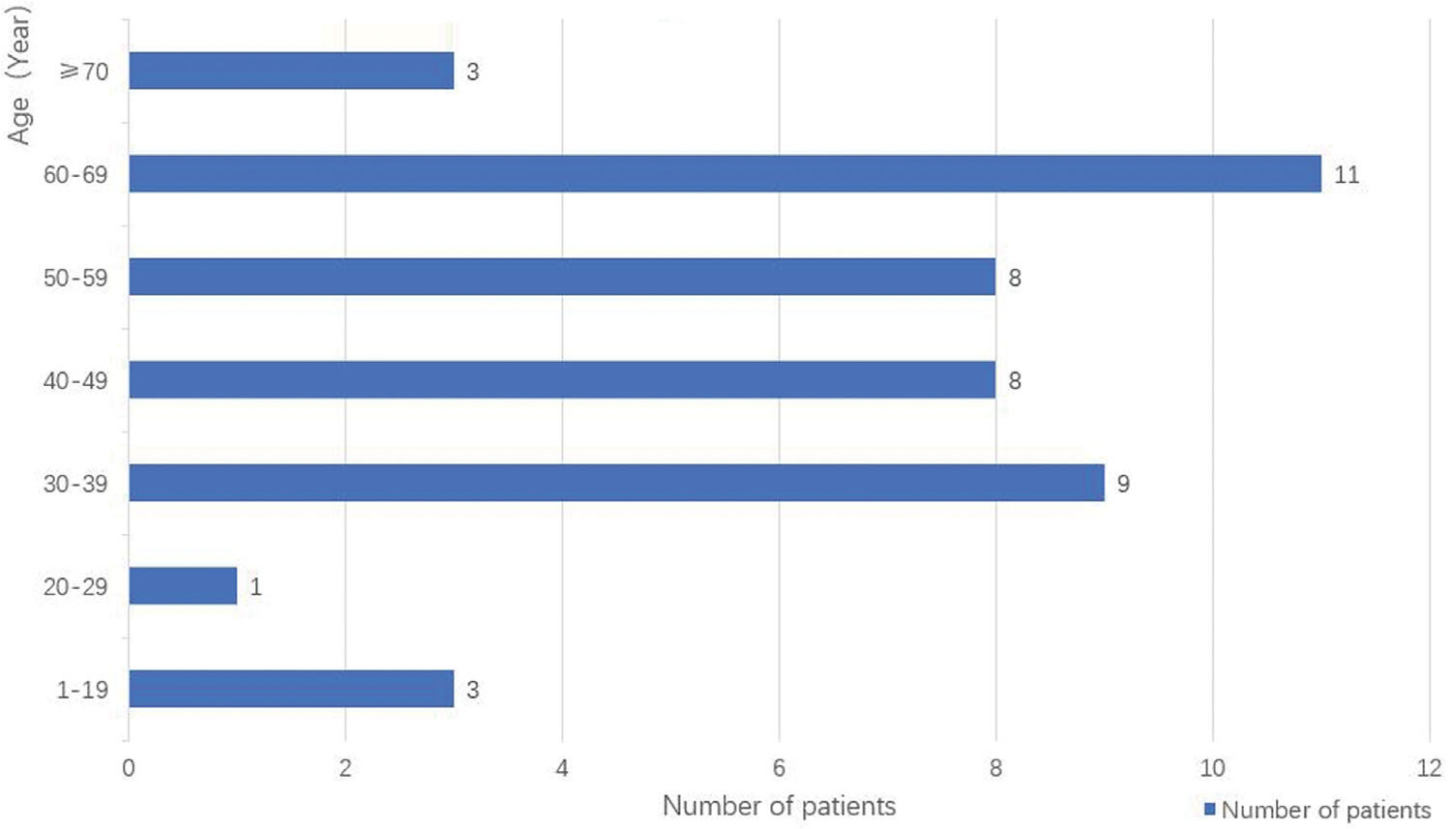
Relationship between the number of patients with paraneoplastic NMOSD and their age. *Note*: The morbidity age of the patients with paraneoplastic NMOSD mostly located in 30–69 years old (36/43, 83.7%), especially 60–69 years old.

In our analysis, 43 AQP4‐positive paraneoplastic NMOSD patients have been included and reported in Tables 2 and S1. The median age is 50 years old. Among these 43 patients, 38 (38/43, 88.4%) patients are female and five (5/43,11.6%) patients are male. The largest number of patients was between 60 and 69 years (Figure 3).

By analyzing the types of tumors in these patients, 19 kinds of tumors were found, involving almost all systems of the human body. One person was reported with three kinds of tumors (Pittock & Lennon, 2008). Breast cancer (10/45, 22.2%) and lung cancer (6/45, 13.3%) were the most common types. We also analyzed other clinical features of the disease, for example, the peak time of disease progression varies from 2 days to 10 months.

Magnetic resonance imaging (MRI) of the spinal cord showed that most of the lesions were located in the cervicothoracic region with patchy gadolinium enhancement. About half of the patients have slight elevation of CSF protein or cells, and about one third of the patients have oligoclonal band (OB), so these features are not specific for the diagnosis of paraneoplastic NMOSD. Five of 43 (5/43, 11.6%) patients tested for CSF AQP4 and they were all positive. Eleven of 43(11/43, 25.6%) patients performed AQP4 test in tumor tissue and seven of them were positive (7/11, 63.6%).

Tumors were not detected in 60.5% of patients until the onset of NMOSD, even 18 months after the onset of NMOSD. Six patients still had NMOSD after tumor surgery (Beauchemin et al., 2018; Bernard‐Valnet et al., 2019; Cai et al., 2016; Etemadifar et al., 2019; Figueroa et al., 2014; Yang et al., 2014; Yuan et al., 2019) and three showed tumor recurrence or metastasis with 3 or 4 years interval between tumor therapy and NOMSD. (Figueroa et al., 2014; Yang et al., 2014; Yuan et al., 2019; Table 3). Two of these six patients relapsed after the first course of NOMSD, and both were with a history of thymoma (Beauchemin et al., 2018; Yang et al., 2014; Table 3). The existing treatment can only delay rather than stop the progress of the disease.

**TABLE 3:** The clinical manifestation of paraneoplastic AQP4‐IgG positive NMOSD with history of tumor surgery

Case no.	Reference	Age (year)/sex	Neurologic deficit	Tumor types	Time from NMOSD to cancer diagnosis. m*	Tumor treatment	Tumor recurrence or metastasis	Treatment	Outcome
1	Junliang Yuan (2019)	60/F	LETM; speech difficulty	Breast cancer	36	Surgery, radiotherapy, chemotherapy	Yes, recurrence	IVMP; IVIGA	Partial recovery
2	Masoud Etemadifar (2019)	34/F	LETM	Meningioma in anterior fossa	1	Surgery	No, but had a fever 1 week before NOMSD	IVMP; rituximab	Partial recovery, no relapse for 2 years
3	Raphaël Bernard‐Valnet (2019)	15/F	Area postrema syndrome	Ovarian teratoma	4	Teratoma ablation	N/A	IVMP; rituximab; chemotherapy	Total recovery, no relapse for 2 years
4	Philippe Beauchemin (2018)	41/F	Unilacteral ON	Thymoma	4	Surgical resection, radiotherapy	N/A	IVMP; azathioprine	Partial recovery, relapse with TM 6 years later
5	Hee Kyung Yang (2014)	55/F	Bilateral ON	Invasive thymoma	72	Thymectomy, radiotherapy, chemotherapy	Yes, metastasis	IVMP, PDN, chemotherapy, azathioprine	Partial recovery, relapse with LETM 13 months later
6	Michelle Figueroa (2014)	48/F	Unilacteral ON; LETM	Hepatic metastasis from a small‐bowel neuroendocrine tumor	72	Surgical resection	Yes, metastasis	IVMP, PE	No progressing

Abbreviations: AQP4, aquaporin‐4; CSF, cerebrospinal fluid; IVIGA, intravenous immunoglobulin; IVMP, intravenous methylprednisolone; LETM, longitudinally extensive myelitis; m*, month; N/A, not applicable; NMOSD, neuromyelitis optica spectrum disorder; ON, optic neuritis; PE, plasma exchange.

## 5 DISCUSSION

In our case, the patient was mainly clinically characterized by myelitis. MRI showed myelitis of more than three spinal segments, and serum AQP4 was positive. According to imaging and other examinations, we can exclude MS, tumor metastasis, and other related differential diagnosis. According to 2015 International NMOSD Diagnostic Criteria (Tan et al., 2016), we preliminarily diagnose the patient as NMOSD. He underwent a surgery 2 years ago because of rectal moderately differentiated adenocarcinoma. PET‐CT this time showed hypermetabolic beside the anastomotic line that was considered as the possibility of recurrence. Moreover, no other immune‐related diseases have been found in this patient and with no history of infection and vaccination, thus we believe that the possibility of paraneoplastic NMOSD is high for this patient. Unfortunately, the patient did not agree to do further colonoscopy, so we could not stain the cancer tissue for AQP4 to verify our inference. The patient experienced an exacerbation of his condition during hospitalization. Though he improved and there were no further episodes of leg weakness after intravenous methylprednisolone (IVMP), intravenous immunoglobulin, and intravenous cyclophosphamide, he was still unable to leave the wheelchair at discharge. After a month of immunomodulation therapy with oral steroids, the weakness of lower extremities got clinical improvement and the spinal MRI showed significantly smaller lesions. We finally diagnosed the patient as AQP4‐positive paraneoplastic NMOSD. Immunotherapy is of great significance but long‐term therapy period should be accepted.

To further explore the relationship between tumor, AQP4, and MNOSD, we searched the previous reported cases and summarized them in Table S1 (Al‐Harbi et al., 2014; Annus et al., 2018; Armağan et al., 2012; Baik et al., 2018; Bernard‐Valnet et al., 2019; Cai et al., 2016; De Santis et al., 2009; Etemadifar et al., 2019; Fang et al., 2019; Figueroa et al., 2014; Frasquet et al., 2013; Jin et al., 2019; Kitazawa et al., 2012; Kon et al., 2017; Liao et al., 2019; Mahale et al., 2019; Mueller et al., 2008; Nakayama‐Ichiyama et al., 2011; Pittock & Lennon, 2008; Verschuur et al., 2015; Wang et al., 2015; Wiener et al., 2018; Yang et al., 2014; Yi & Park, 2020; Yuan et al., 2019) and did further quantitative analysis in Table 2. This systematic analysis allows for tumor screening as part of the etiological screening of the tumor high‐risk subset of NMOSD patients. NMOSD is most common among young people (between 30 and 50 years) and there is a female predominance (3:1–9:1). The median age of our AQP4‐positive paraneoplastic NMOSD patients is 50 years that is associated to the late age of tumor onset. The occurrence of AQP4‐positive NMOSD was associated with a wide range of cancer types, especially breast cancer and lung cancer. However, due to the limited number of cases, we cannot classify the types of tumors with high incidence in each age group. Other clinical features of paraneoplastic NMOSD are almost consistent with that of NMOSD originated from other causes. We speculated that for AQP4‐positive NMOSD patients, especially the elderly, if other immunological factors are excluded, cancer screening should be taken under consideration that is of great significance for disease prognosis. However, for different age groups, the types of tumors to be screened may be different, which requires more clinical data.

We observed refractory hyponatremia in our patient. Hyponatremia is a common electrolyte disorder in patients with neurological disorders and has also been reported in patients with NMOSD (Pu et al., 2015). In NMOSD patients, syndrome of inappropriate antidiuretic hormone secretion (SIADH) is the most common cause of hyponatremia, accounting for about 14.6%–16% (Iorio et al., 2011; Pu et al., 2015).The hypothalamus is the area of high expression of AQP4. Immune‐mediated hypothalamic supraventricular nucleus and paraventricular nucleus lesions can lead to abnormal secretion of antidiuretic hormone (ADH), thus causing SIADH. Circumventricular organs (CVOs) that are located around the third and fourth ventricles with abundant AQP4 expression are the target of NMOSD. It plays a number of important functions through its connections with the hypothalamus and brainstem, including the regulation of sodium and water, so CVOs lesion in patients with NMOSD may lead to SIADH. Another cause of hyponatremia is known as cerebral salt wasting syndrome (CSWS), which is caused by neurological disorders that lead to decreased sympathetic nerve signals to the kidneys or increased brain natriuretic peptide in the blood, resulting in excessive sodium excretion from normal functioning kidneys. Spinal cord injury, a common lesion in NMOSD, can lead to autonomic nervous system damage—sympathetic dysfunction of blood vessels, leading to hypovolemia and hypotension, which promotes the secretion of antidiuretic hormone and increase renal water retention. Retention of renal fluid leads to dilutional hyponatremia. Furthermore, spinal cord injury can cause damage to descending renal sympathetic nerve pathways that impair renal sodium preservation and water excretion. Therefore, spinal cord injury can lead to the reduction of kidney to retain sodium ions, resulting in CSWS. In our case, the patient had immunologic spinal cord injury and no abnormality was found in brain MRI. Therefore, the cause of hyponatremia was considered to be spinal CSWS. With the recovery of spinal cord injury, hyponatremia can recover spontaneously, so only supportive treatment was given to this patient. Intractable hyponatremia was corrected 1 month later with the partial recovery of the spinal cord lesion. For the treatment of hyponatremia associated with nervous system diseases, the most important thing is to identify the cause and symptomatic treatment.

AQP4 is an aquaporin protein expressed on the end feet of astrocytes in the brain, spinal cord, and optic nerve, especially in the areas adjacent to the CSF (Rash et al., 1998). As one of the possible mechanisms of morbidity, AQP4‐Ab‐mediated humoral immunity is gradually accepted by people. As an aquaporin, AQP4 is not only widely expressed in the central nervous system, but also in nonnervous tissues, including skeletal muscle cells, lung airway cells, gut epithelium, gastric parietal cells, kidney collecting duct cells, placenta, glioblastomas, olfactory epithelial cells, and so on (Verkman et al., 2014). AQP4 was found to be highly expressed in a variety of tumor cells including lung cancer and breast cancer and can promote tumor progression, invasion, and metastasis (Li et al., 2016; Warth et al., 2011). The expression of AQP4 was low in breast cancer and gastric cancer, but high in lung cancer, meningiomas, and thyroid carcinoma (Papadopoulos & Saadoun, 2015). Paradoxically, in our analysis, breast cancer and lung cancer are the most common tumor types in patients with AQP4‐positive paraneoplastic NMOSD, which may be associated with their high incidence. In a 10‐year retrospective Mayo study of AQP4‐positive paraneoplastic NMOSD, 93% of AQP4 ‐positive patients had symptoms of NMOSD (Pittock & Lennon, 2008). A study from Cleveland Clinic showed the presence of concurrent tumors in 15% of patients with AQP4‐positive NMOSD (Ontaneda & Fox, 2014). Another study showed that 27% of patients with AQP4‐positive neuromyelitis optica were diagnosed with cancer shortly before and after diagnosis (Pittock & Lennon, 2008). Also, in this study, two patients with seropositive NMO‐IgG antibodies did not show symptoms of NMOSD, but were found to have tumors (one with breast cancer and one with lung cancer; Pittock & Lennon, 2008). In our analysis, 11 patients (Annus et al., 2018; Baik et al., 2018; Beauchemin et al., 2018; Bernard‐Valnet et al., 2019; Figueroa et al., 2014; Jin et al., 2019; Kon et al., 2017; Nakayama‐Ichiyama et al., 2011; Verschuur et al., 2015) performed AQP4 test in tumor tissue and seven of them are positive (7/11, 63.6%; Baik et al., 2018; Beauchemin et al., 2018; Bernard‐Valnet et al., 2019; Figueroa et al., 2014; Jin et al., 2019), four of them are negative (4/11, 36.3%; Annus et al., 2018; Kon et al., 2017; Nakayama‐Ichiyama et al., 2011; Verschuur et al., 2015). The relatively high negative rate could be related to the limited size of tumor tissue sample. It is worth noticing that 74.4% (32/43) of serum AQP4‐positive patients did not perform tumor tissue AQP4 test, which should be improved in our future diagnosis process. We believe that the detection of AQP4 antibody in tumor tissue is the most direct method to confirm the origin of serum AQP4 antibody and the etiology of NMOSD. If the serum AQP4 is positive, it is best to do further AQP4 immunostaining of tumor tissue. In NMOSD, the damage caused by AQP4 antibody often occurs around blood vessels, first manifested by complement‐mediated loss of a large number of astrocytes, followed by the damage of oligodendrocytes and neurons, ultimately lead to demyelination and severe neuronal damage (Jarius et al., 2020). Unlike other CNS neuroimmune diseases, NMOSD lacks intrathecal antibody synthesis, and the concentration of AQP4 in plasma is more than 500 times than that in CSF, indicating that AQP4 is formed peripherally and enters the central nervous system (Jarius et al., 2010). And before the morbidity of NMOSD, there are often incentives such as infection, which may temporarily increase the permeability of the blood–brain barrier in the optic nerve or spinal cord, thus promoting the circulation of AQP4 antibodies into the central nervous system (Papadopoulos & Verkman, 2012). Serum AQP4 in four patients were reexamined after treatment, and three of them were AQP4 negative. One patient was still positive, but the level of AQP4 was decreased. Serum AQP4 can be used as a means of follow‐up and preventing recurrence.

The clinical management of patients with paraneoplastic syndromes usually involves four aspects: recognition of the disease as a paraneoplastic syndrome, identification of the associated tumor, treatment of the tumor, and suppression of the autoimmune response that causes neurologic damage (Dropcho, 2005). Accordingly, we believe that the management of paraneoplastic NMOSD should also include the above four steps. First, clinical characteristics should meet the diagnostic criteria of NMOSD, then the cause screening should be followed. If the immune factors are excluded and the patient is with high risk of cancer, neoplasm‐related screening should be performed. Whole‐body 18 FDG‐PET may be useful for locating the occult cancer. Paraneoplastic syndrome is the result of remote effect of a malignant neoplasm that occurs in the early stage of tumor, so multiple tumor screenings may be required. In more than 80% of patients, the tumor is found months to years after the onset of neurological symptoms (Honnorat & Antoine, 2007). In our statistics, tumors were not detected in 60.5% of patients until the onset of NOMSD, or even 18 months after the onset of NMOSD. There is no consensus that AQP4‐positive NMOSD should be monitored regularly for cancer if immunological factors are excluded, but we believe that older patients with associated high‐risk factors should be monitored. In some patients, the onset of symptoms of NMOSD lags behind the discovery of the tumor, and NMOSD occurs even after tumor treatment (Beauchemin et al., 2018; Bernard‐Valnet et al., 2019; Cai et al., 2016; Etemadifar et al., 2019; Figueroa et al., 2014; Yang et al., 2014; Yuan et al., 2019). Therefore, regular monitoring of serum AQP4 in AQP4‐positive paraneoplastic NMOSD patients may contribute to early detection of tumor recurrence or metastasis.

If the diagnosis of paraneoplastic NMOSD is definite, the core of treatment is about the primary tumor, which avoids further deterioration of the disease and is conducive to recovery. Immunomodulatory therapies are also of great significance in addition to cancer therapy. NMOSD treatment includes two steps: acute attack treatment and long‐term maintenance treatment (Kessler et al., 2016). Acute attacks of NMOSD can lead to varying degrees of inflammatory injuries. The purpose of acute treatment is to alleviate the acute inflammatory attack and minimize CNS damage (Kessler et al., 2016). IVMP is usually the first choice of treatment in the acute phase, plasma exchange and IVIG are used as an escalatory treatment for those patients with inadequate response to IVMP (Bruscolini et al., 2019; Kessler et al., 2016). Maintenance treatment is aimed at better alleviation of symptoms and prevention of recurrence. The immunotherapy mainly includes immunosuppressive drugs in combination with low‐dose corticosteroids (Holmøy et al., 2020). Several new drugs, such as eculizumab, satralizumab, and inebilizumab, have demonstrated efficacy in phase III clinical trials targeting the maintenance phase of NMOSD (Valencia‐Sanchez & Wingerchuk, 2021). Theoretically, there is a possibility that immunosuppression may lead to tumor progression, but there is no clear evidence, and immunosuppression may have some benefits in improving nervous system damage, so there is no consensus on whether immunosuppressive agents should be used in patients with paraneoplastic NMOSD. Paraneoplastic syndromes‐associated antineuronal antigens are divided into two types (Höftberger et al., 2015). One exists in nerve cells, and its toxicity is mediated by cytotoxic T cells, often leading to irreversible cell damage or death. Another kind of antigen is located on the cell surface or synapse, which directly mediates nerve injury through direct antigen–antibody reaction. AQP4 is water channel protein widely expressed in the astrocyte cell plasma membrane. AQP4‐positive NMOSD could lead to central or peripheral nerve injury by the binding of AQP4‐specific autoantibody to APQ4 on the plasma membrane of astrocytes. Therefore, the most direct therapeutic measure is the blocking therapy, that is, to prevent AQP4‐specific autoantibodies from binding to AQP4 on astrocyte membranes. This molecule therapy is something to look forward to.

## 6 CONCLUSION

Here, we reported a case of AQP4‐positive paraneoplastic NMOSD related with recurrent rectal cancer and perform a retrospective analysis of previously reported cases. NMOSD is a female predominance disease with typical onset age in the third to fourth decades of life. For the elderly, NMOSD could just be the presentation in nervous system, indicating some underlying tumors. For the elderly, people at high risk of cancer and patients with poor treatment response, tumors should be suspected as an underlying cause and screened thoroughly. Paraneoplastic syndromes caused by tumor recurrence and metastasis need to be fully considered in patients with a history of cancer and clinical manifestation of NMOSD. Early detection of underlying tumors and combination of surgery and immunotherapy are critical for a good prognosis. More data are needed to further differentiate the characteristics of NMOSD caused by different types of tumors, so as to achieve more targeted cancer screening.

## CONFLICT OF INTEREST

The authors declare no conflict of interest.

### FUNDING

This work was supported by a grant from the National Natural Science Foundation of China (82071351).

### PEER REVIEW

The peer review history for this article is available at https://publons.com/publon/10.1002/brb3.2282

## Supporting information

SUPPORTING INFORMATION

Table S1. Characteristics of patients with AQP4‐positive paraneoplastic NMOSD
