# Supplementary material for: AQP4‐IgG positive paraneoplastic NMOSD: A case report and review
Source: Brain Behav. 2021 Sep 14;11(10):e2282. doi: 10.1002/brb3.2282 (PMC8553315; doi:10.1002/brb3.2282)
Supplement: Supplementary file 1 — SUPPORTING INFORMATION [file BRB3-11-e2282-s001.docx]

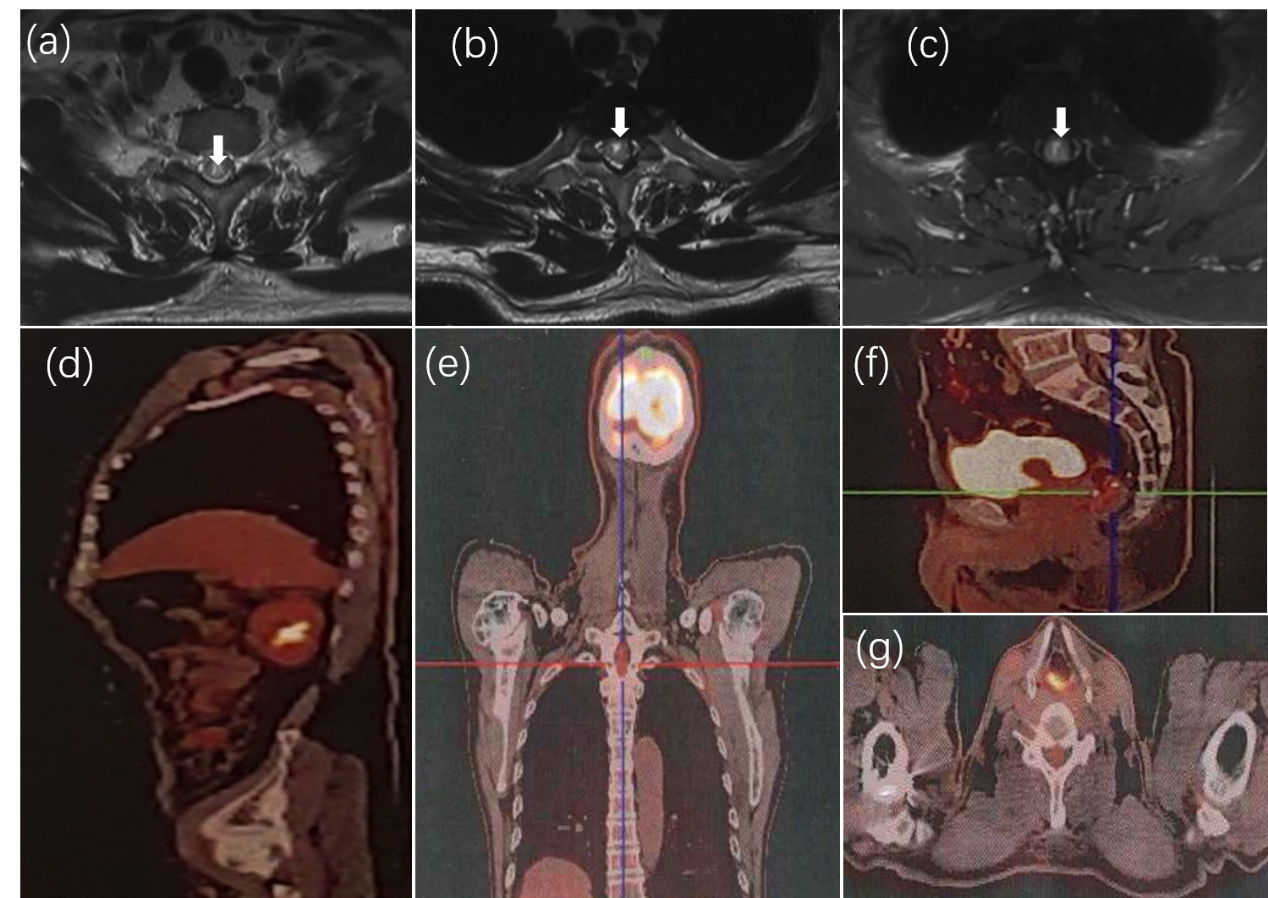


**Supplemental Figures**

Axial MRI T2 image of cervical spine(a); Axial MRI T2 image of thoracic spine(b); Axial MRI T1 image of the thoracic spine postinfusion of gadolinium(c); Coronal cervicothoracic region PET/CT images (e); Axial thoracic region PET/CT image(g); Intestinal wall thickening with hypermetabolism in rectal anastomotic area(d/f). A indicates anterior; H, head.
