# Supplementary material for: AQP4‐IgG positive paraneoplastic NMOSD: A case report and review
Source: Brain Behav. 2021 Sep 14;11(10):e2282. doi: 10.1002/brb3.2282 (PMC8553315; doi:10.1002/brb3.2282)
Supplement: Supplementary file 2 — Table S1. Characteristics of patients with AQP4‐positive paraneoplastic NMOSD [file BRB3-11-e2282-s002.docx]

|  |  |  |  |  | Supplemental Table 1 Characteristics of Patients with AQP4-positive Paraneoplastic NMOSD | | | | | | | | | | |
| --- | --- | --- | --- | --- | --- | --- | --- | --- | --- | --- | --- | --- | --- | --- | --- |
| Case No. | Reference | Age(yr)/Sex | Time of Onset | Disease  Progression  Period | Neurologic Deficit | Neuroimaging Location | CSF Routine (cell: /ul, protein: mg/dl, Glu: mg/dl) | Oligoclonal Band Test | Onset AQP4 (Serum/CSF/  Cancer Tissue） | Follow-up AQP4 | Other Immune System Disorders | Most Recent Cancer | Time From NMOSD to Cancer Diagnosis. m* | Treatment | Outcome |
|  |  |  |  |  |  |  |  |  |  |  |  |  |  |  |  |
| 1 | SangHak Yi（2020） | 54/F | presentation | 10 days | LETM;  Unilacteral ON | C3-T2,T2 hyperintense signals,  Gd contrast: enhancement | WBC 0, Protein 48.6, Glu: 80 | CSF: Neg | Serum: Pos  CSF: N/A  Cancer: N/A | N/A | N/A | Bladder Urothelial Carcinoma | -1 | Acute; IVMP; Radical Cystectomy.  Chronic: Oral prednisolone | LETM: PR; ON: CR; No relapse for 2 years |
| 2 | Junliang Yuan (2019) | 60/F | presentation | 10 days | LETM;  Speech  Difficulty | Brain MRI: splenium and the body of corpus callosum, midbrain; T2 hyperintense signals; slight contrast enhancement; Cervical MRI(C3-T5): T2 hyperintense lesions Thoracic MRI(T7-T12): T2 hyperintense lesions, slight contrast enhancement PET-CT: the right breast cancer | WBC 38; Protein 189; IgG 16.30 mg/dl; Ig G index 0.9; MBP (serum and CSF): positive | Serum: Pos; CSF: Pos | Serum: Pos  CSF: Pos  Cancer: N/A | N/A | N/A | Breast Cancer | 36 | Acute: IVMP; IVIGA | PR |
| 3 | Rohan R Mahale (2019) | 67/F | presentation | 3 days | LETM | D1 to D10 vertebral levels: T2 hyperintense signal, contrast enhancement | no pleocytosis,  no malignant cells;  Protein: 84;  Glu: 76 | N/A | Serum: Pos CSF: N/A Cancer: N/A | N/A | N/A | Renal Cell Carcinoma | 0 | Acute: IVMP; PE | PR |
| 4 | Liao Weijing（2019） | 35/F | presentation | 3 months | LETM; Unilacteral ON | C3-C4, T2 hyperintense signal | Normal | N/A | Serum: Pos CSF: N/A Cancer: N/A | Neg, after surgery | N/A | Breast Cancer; invasive ductal carcinoma | -14 | Acute: IVMP; Surgery;  Chronic: Azathioprine | LETM: CR; ON: PR; Reoccur for 3 times before cancer diagnosis |
| 5 | Haiqiang Jin（2019） | 31/F | presentation | 1 months | LETM;  Fever | C2-T4, with gadolinium enhancement | Pleocytosis 135, Protein 1346.7, Glu 3.1. | Serum: Pos; CSF: Pos | Serum: Pos  CSF: Pos;  Cancer: Pos | N/A | N/A | Renal Clear Cell Carcinoma | -1 | Acute: IVIG, IVMP, Predisone, Tacrolimus; Surgery | Almost CR |
| 6 | Fang Wei (2019) | 61/F | Relapse (first time--about 1.5 years ago) | 10 days | LETM | Thoracic spinal cord for multiple segments, T2 hyperintense signals | Pleocytosis: 26, Protein :59.0,  Glu: normal | CSF: Neg | Serum: Pos CSF: N/A Cancer: N/A | N/A | N/A | Lung Adenocarcinoma and Intraductal Papillary Mucinous Neoplasm | 0 | Acute: IVIG, IVMP, Prednisone | Almost CR after surgery |
| 7 | Masoud Etemadifar（2019） | 34/F | N/A | N/A | LETM | T6-T9 | Normal | N/A | Serum: Pos CSF: N/A Cancer: N/A | N/A | N/A | Meningioma in anterior fossa | 1 | Acute: IVMP;  Chronic: Rituximab | PR and no relapse for 2 years |
| 8 | Raphaël Bernard-Valnet (2019) | 15/F | presentation | 5 weeks | Area Postrema Syndrome | Brainstem, Cervical (C2, C4-C5, C6-C7), T2 hyperintense signal | Pleocytosis: 65 | CSF: Neg | Serum: Pos;  CSF: Pos  Cancer: Pos | N/A | N/A | Ovarian Teratoma | 4 | Acute: IVMP;  Chronic: Rituximab; Chemotherapy | CR and no relapse for 2 years |
| 9 |  | 21/F | presentation | N/A | LETM;  Area Postrema Syndrome; | C3-C4, T8 and conus | Pleocytosis: 87 | CSF: Pos | Serum: Pos;  CSF: Pos  Cancer: Pos | N/A | N/A | Ovarian Teratoma | -1 | IVMP; PE | 2 optic neuritis episodes before teratoma ablation; no relapse |
| 10 | Daniel C. Wiener (2018) | 62/M | presentation | N/A | LETM | Throughout the cervical and thoracic spinal cord | WBC 6, Protein 68.3, Glu: Normal | CSF: Neg | Serum: Pos CSF: N/A Cancer: N/A | Neg | N/A | Esophageal Adenocarcinoma | 0（NMOSD before cancer） | Acute: IVMP, PE, Surgery | PR |
| 11 | Kyoung Won Baik (2018) | 37/F | presentation | 3 months | LETM; Unilacteral ON | C2-T7, T2 hyperintense; with gadolinium enhancement | No malignant cells, Pleocytosis 60 (98% mononuclear cells),  Protein: 138.2 | CSF: Neg | Serum: Pos;  CSF: N/A Neck lymph nodes: Pos | N/A | N/A | Lung Adenocarcinoma | N/A (cancer before NMOSD) | Acute: IVMP  Chronic: Azathioprine; Chemotherapy | PR and no relapse |
| 12 | Philippe Beauchemin (2018) | 54/F | presentation | N/A | Area Postrema Syndrome; | Medulla to C5 | WBC 96 (38% neutrophils, 62% lymphocytes), Protein: 152.5 | CSF: Neg | Serum: Pos;  CSF: N/A;  Cancer: Pos | N/A | Hypothyroidism | Ovarian serous carcinoma | -18 | Acute: IVMP, PE and  Chemotherapy Chronic: Azathioprine | PR and no relapse for 2 years. |
| 13 |  | 41/F | presentation | N/A | Unilacteral ON | Orbits MRI | N/A | N/A | Serum: Pos;  CSF: N/A;  Cancer: Pos | N/A | Hepatitis B,  Sjogren’s  disease | Thymoma | 4 | Acute: IVMP  Chronic: Azathioprine | PR and 1 TM 6 years later |
| 14 | Ádám Annus (2017) | 66/F | presentation | N/A | LETM; Unilacteral ON | T4-T10 | Protein 85;  WBC 15 | CSF: Pos | Serum: Pos; CSF: Pos;  Cancer: Neg | N/A | MDS;  refractory anaemia | Lung Squamous cell carcinoma | 0 | Acute: IVMP; IVIGA | Die |
| 15 | Tomoya Kon (2017) | 70/F | presentation | 1 months | LETM | C4-C6: T2 hyperintense, T1 gadolinium enhancement | Normal | N/A | Serum: Pos;  CSF: N/A Cancer: Neg | Neg | N/A | Esophageal Squamous Cell Cancer (T3N1M0) | -1 | Acute: IVMP; Adiation Therapy; Chemotherapy; | PR and no relapse for 1 year |
| 16 | Gang Cai（2016） | 17/F | presentation | 1 months | LETM;  Area Postrema Syndrome; Bilateral ON | Medulla and C1-C2, T2 hyperintese, without enhancement | WBC and Protein:  normal | N/A | Serum: Pos CSF: N/A Cancer: N/A | N/A | N/A | Papillary Thyroid  Carcinoma | -1 | Acute: IVMP. Thyroidectomy Chronic: Low-dose prednisone | PR and relapse 10 months later |
| 17 |  | 35/M | presentation | 3 days | Bilateral ON | Swelling and enlargement of bilateral optic nerves | WBC and Protein:  normal | N/A | Serum: Pos CSF: N/A Cancer: N/A | N/A | N/A | Acute myeloid leukemia (M2) | 10 | IVMP, Oral PDN,  Chemotherapy | Slightly improved, relapse 8 months later |
| 18 |  | 43/F | presentation | 1 week | Area Postrema Syndrome | From the mesencephalon to the medulla, with Gd enhancement | WBC and Protein:  normal | N/A | Serum: Pos CSF: N/A Cancer: N/A | N/A | N/A | Breast carcinoma | -3 | IVMP, Oral PDN, Mastectomy, Chemotherapy | CR and no relapses for 3 years |
| 19 | Yujie Wang (2015) | 50/F | presentation | 6 months | Progressive Painful  Left Eye Vision Loss | Orbit, brain, and spine MRI: normal | CSF: normal | CSF: Neg | Serum: Pos CSF: N/A Cancer: N/A | N/A | Hypothyroidism and gastric ulcer; Elevated anticardiolipin IgM | Marginal zone lymphoma | 12 | IVMP | CR and no relapse for 9 months |
| 20 | Constant Valentijn Michiel Verschuur (2015) | 39/F | presentation | 3 weeks | Area Postrema Syndrome; Blurred  and Hazy Vision | right side of the medulla oblongata and the left side of the mesencephalon | 375 leucocytes, Without tumor cells | N/A | Serum: Pos;  CSF: N/A Cancer: Neg | N/A | N/A | Lung Adenocarcinoma | -0.5 | N/A | Deteriorated and Die |
| 21 | Hee Kyung Yang (2014) | 55/F | presentation | 5 months | Bilateral ON | Both optic nerves: | Normal | CSF: Neg | Serum: Pos CSF: N/A Cancer: N/A | N/A | N/A | Invasive thymoma | 72 | IVMP, PDN, Chemotherapy, Azathioprine | PR and relapse with LETM |
| 22 | Michelle Figueroa（2014） | 48/F | presentation | 10 months | LETM;  Unilacteral ON | C6-C4, T2 hyperintensity; T3-T4 Gd enhancement | Normal | CSF: Neg | Serum: Pos;  CSF: N/A  Cancer: Pos | N/A | Sticky platelet syndrome, Antiphospholipid syndrome, and increased von Willebrand factor activity; ANA 1:160, double-stranded DNA 1:80 | Hepatic metastasis from a small-bowel neuroendocrine tumor | 72 | IVMP, PE | No progressing |
| 23 | Talal Al-Harbi (2014) | 38/F | presentation | 2 months | LETM;  Area Postrema Syndrome;  ON | Medulla to C2, with enhancement | Normal | N/A | Serum: Pos CSF: N/A Cancer: N/A | N/A | N/A | Stomach carcinoid tumor | -2 | Acute: IVMP, IVIGA; PDN Gastric ablation  Chronic: Azathioprine, and Vitamin B12 | MRI: significant regression |
| 24 | Marina Frasquet (2013) | 42/F | presentation | 2 days | LETM;  Area Postrema Syndrome | Lower brainstem to the conus medullaris, with enhancement | WBC 260, protein 80.5, normal glucose | CSF: Pos | Serum: Pos CSF: N/A Cancer: N/A | N/A | N/A | Ovarian mature cystic teratoma | 0 | IVMP, IVIG, PE, Rituximab | PR and no relapse for 1 year |
| 25 | Yu Kitazawa (2012) | 87/M | relapse(first time--about 3 months ago) | N/A | LETM;  Area Postrema Syndrome;  ON | T4-T10 | Protein: slightly increase | CSF: Neg | Serum: Pos CSF: N/A Cancer: N/A | N/A | N/A | Prostate Adenocarcinoma | 3 | IVMP | ON: PR; LETM: NR |
| 26 | Haydar Armağan (2012) | 62/F | presentation | N/A | LETM;  Area Postrema Syndrome | C2-C6, T2 hyperintense, with patchy contrast enhancement； | Acellular, Protein 78, Glu normal | CSF: Neg | Serum: Pos CSF: N/A Cancer: N/A | Serum:  Neg | N/A | Breast cancer，stage 3 invasive ductal carcinoma | -3 | IVMP, Oral steroid, Azathioprine, Radical mastectomy, Chemotherapy | PR |
| 27 | Shoko Nakayama-Ichiyama（2011） | 57/F | presentation | N/A | LETM | C5 to T4, T2 hyperintense | Pleocytosis 44, Glu: normal, Protein 49; | CSF: Neg | Serum: Pos； CSF: N/A Abnormal cells: Neg | Serum: Pos, but decrease | SIADH | Mature B-cell neoplasm | N/A (NMOSD before cancer) | IVMP; Cetamethasone; Chemotherapy | PR |
| 28 | Giuseppe De Santi  （2009） | 63/F | presentation | 3 months | LETM;  Bilateral ON | Lower cervical region (C5–C6, C7) and upper dorsal region (D1, D2), T2 hyperintense | Protein 65 | CSF: Pos | Serum: Pos CSF: N/A Cancer: N/A | N/A | N/A | Lung cancer, non-small cell carcinoma. | -3 | IVMP, PE, Chemotherapy | Die |
| 29 | Sabine Mueller（2008） | 63/F | relapse(LETM,15 months ago ) | 2 days | LETM | C5 to T10, Patchy enhancement was noted from T6 to T9 | N/A | N/A | Serum: Pos CSF: N/A Cancer: N/A | N/A | N/A | Metastatic breast cancer | 24 | IVMP, Chemotherapy | PR and die |
| 30 | Sean J. Pittock（2008） | 36/F | N/A | N/A | NMO | N/A | N/A | N/A | Serum: Pos CSF: N/A Cancer: N/A | N/A | N/A | Breast carcinoma | 5 | N/A | N/A |
| 31 |  | 63/F | N/A | N/A | LETM | N/A | N/A | N/A | Serum: Pos CSF: N/A Cancer: N/A | N/A | N/A | Breast carcinoma (infiltrating ductal) | -14 | N/A | N/A |
| 32 |  | 66/F | N/A | N/A | LETM | N/A | N/A | N/A | Serum: Pos CSF: N/A Cancer: N/A | N/A | N/A | Lung carcinoma | Anteceded rLETM (interval unknown) | N/A | N/A |
| 33 |  | 44/F | N/A | N/A | NMO | N/A | N/A | N/A | Serum: Pos CSF: N/A Cancer: N/A | N/A | N/A | Thymic carcinoma | -3 | N/A | N/A |
| 34 |  | 18/F | relapse | N/A | rLETM | N/A | N/A | N/A | Serum: Pos CSF: N/A Cancer: N/A | N/A | N/A | Cervical carcinoma | 3 | N/A | N/A |
| 35 |  | 70/M | relapse | N/A | rLETM | N/A | N/A | N/A | Serum: Pos CSF: N/A Cancer: N/A | N/A | N/A | Seminoma(metastasis); Bladder carcinoma); B-cell lymphoma | -288/-140/-18 | N/A | N/A |
| 36 |  | 61/F | relapse | N/A | rLETM | N/A | N/A | N/A | Serum: Pos CSF: N/A Cancer: N/A | N/A | N/A | Monoclonal gammopathy | Anteceded rLETM (interval unknown) | N/A | N/A |
| 37 |  | 49/F | relapse | N/A | rLETM | N/A | N/A | N/A | Serum: Pos CSF: N/A Cancer: N/A | N/A | N/A | Breast carcinoma | -6 | N/A | N/A |
| 38 |  | 55/F | N/A | N/A | NMO | N/A | N/A | N/A | Serum: Pos CSF: N/A Cancer: N/A | N/A | N/A | Breast carcinoma | 60 | N/A | N/A |
| 39 |  | 53/F | N/A | N/A | NMO | N/A | N/A | N/A | Serum: Pos CSF: N/A Cancer: N/A | N/A | N/A | Thyroid (Hurthle cell) | -12 | N/A | N/A |
| 40 |  | 31/F | N/A | N/A | NMO | N/A | N/A | N/A | Serum: Pos CSF: N/A Cancer: N/A | N/A | N/A | Pituitary somatotropinoma | -3 | N/A | N/A |
| 41 |  | 51/F | N/A | N/A | NMO | N/A | N/A | N/A | Serum: Pos CSF: N/A Cancer: N/A | N/A | N/A | Breast carcinoma | 180 | N/A | N/A |
| 42 |  | 47/F | N/A | N/A | NMO | N/A | N/A | N/A | Serum: Pos CSF: N/A Cancer: N/A | N/A | N/A | B-cell lymphoma | 55 | N/A | N/A |
| 43 |  | 40/M | N/A | N/A | NMO | N/A | N/A | N/A | Serum: Pos CSF: N/A Cancer: N/A | N/A | N/A | Monoclonal gammopathy | 11 | N/A | N/A |
| NMOSD: Neuromyelitis Optica Spectrum Disorders; LETM: Longitudinally Extensive Myelitis; rLETM: Recurrent Longitudinally Extensive Myelitis; ON: Optic Neuritis; rON: Recurrent Optic Neuritis; Bilateral ON: Bilateral Optic Neuritis; Unilateral ON: Unilateral Optic Neuritis; TM: Transverse Myelitis; CSF: Cerebrospinal Fluid; IgG index: Immunoglobulin G index; OBs: Oligoclonal Bands; AQP4: Aquaporin-4; IVMP: Intravenous methylprednisolone; PLEX: Plasmapheresis; Gd: Gadolinium; IVIGA: Intravenous Immunoglobulin; CTX: Cyclophosphamide; PE: Plasma Exchange; Neg: Negative; Pos: Positive; N/A: Not Applicable;CR: Complete Response; PR: Partial Response; NR: No Response; TM: Transverse Myelitis; MBP: Myelin Basic Protein | | | | | | | | | | | | | | | |
|  | | | | | | | | |  |  |  |  |  |  |  |
|  |  |  |  |  |  |  |  |  |  |  |  |  |  |  |  |
